# Evaluation of thyroid nodules in the Brazilian Public Health Care System, Supplementary Health System, and Private Health System in the northeastern region of the State of São Paulo

**DOI:** 10.20945/2359-3997000000294

**Published:** 2020-10-09

**Authors:** Ana Paula Figueiredo Engler Goulart, Eduardo Ruas Martins Batista, Marcos Gomes Figueira, Patrícia Künzle Ribeiro Magalhães, Léa Maria Zanini Maciel

**Affiliations:** 1 Universidade de São Paulo Faculdade de Medicina de Ribeirão Preto Departamento de Clínica Médica Ribeirão Preto SP Brasil Divisão de Endocrinologia, Departamento de Clínica Médica, Faculdade de Medicina de Ribeirão Preto da Universidade de São Paulo, Ribeirão Preto, SP, Brasil; 2 Soares Patologia e Citopatologia Franca SP Brasil Soares Patologia e Citopatologia, Franca, SP, Brasil

**Keywords:** Thyroid nodule, fine-needle aspiration biopsy, cytological techniques, diagnostic techniques, endocrine, delivery of healthcare

## Abstract

**Objective:**

To obtain data about the evaluation of thyroid nodules (TNs) in the northeastern of the State of São Paulo, compared by health care type, and measure the performance of cytology as a screening test for thyroid cancer (TC).

**Subjects and methods:**

We collected data of 597 patients treated in the Brazilian public health care system (SUS), supplementary health (SH) and in private health system (PHS) in 2014. A total of 803 TNs were aspirated, and 125 patients underwent surgery.

**Results:**

The distribution of all cytologic results according to the Bethesda system was: I, 135 (16.8%); II, 475 (59.2%); III, 107 (13.3%); IV, 32 (4.0%); V, 20 (2.5%); VI, 34 (4.2%). The time between cytologic analysis and surgery was longer in the SUS than in the SH for TNs in general (p < 0.001) and for TNs with Bethesda V and VI cytology (p = 0.01). The sizes of the TNs and resected malignant TNs was larger in the SUS than in the SH (p = 0.001 and p = 0.02, respectively). The number of PHS surgeries was too small and was not compared. The prevalence of TC was 9.2% and 23.6% of them were treated in the SUS. Cytology showed a 93.6% sensitivity, 95.8% specificity, and 94.7% accuracy when Bethesda III and IV were excluded.

**Conclusion:**

Cytology was a good screening test for TC categories Bethesda II, V, and VI. The differences between the SUS and SH indicate a need for improved access to consultations and specialized tests in the SUS.

## INTRODUCTION

Thyroid nodules (TNs) represent a common clinical scenario and are detected in approximately 5% of women and 1% of men on cervical palpation ( 1 ) and up to 19–68% of the individuals on cervical ultrasound (US) ( 1 , 2 ).

The evaluation of patients with TNs begins with clinical assessment followed by laboratory tests including serum TSH measurement, cervical US, cytologic analysis, and, eventually, molecular tests ( 1 , 3 , 4 ).

Cervical US is an essential test for the evaluation of TNs since it is accessible, noninvasive, and cost-effective ( 5 ). Since 2009, specialty societies have proposed algorithms to stratify the risk of malignancy based on US in order to direct the evaluation of TNs toward fine-needle aspiration biopsy (FNAB) or clinical monitoring ( 1 , 6 – 8 ).

Cytological analysis is the method with the greatest accuracy and cost-effectiveness for the assessment of TNs ( 9 ), and the results are reported according to the Bethesda classification system ( 10 , 11 ).

Thyroid cancer (TC) can occur in 5 to 15% of all TNs ( 1 , 2 ). The two major forms of differentiated thyroid cancer (DTC) are papillary carcinoma (PTC) and follicular carcinoma (FTC). PTC represents approximately 85% of all TCs. FTC represents about 2 to 5% and are recognized as minimally invasive and widely invasive according to the invasion pattern ( 1 ). Undifferentiated carcinomas and anaplastic carcinomas are more aggressive and rare. Medullary thyroid carcinomas (MTC) represent about 3–5% of all TCs, differing from DTCs in biological behavior ( 12 ).

Data from the United States, Europe, Oceania, South America and Asia have confirmed a progressive and generalized increase in TC rate, with PTCs mainly accounting for this increase ( 13 ). In Brazil, data from the National Cancer Institute (INCA) estimate 1570 new cases of TC in men and 8040 in women in the 2018-2019 biennium, placing TC as the 5^th^ and 13^th^ cause of cancer in women and men, respectively ( 14 ). Two Brazilian epidemiological studies conducted in the Greater Florianópolis (2000 to 2005 data) and Greater São Paulo (1997 to 2008 data) areas have shown an increased incidence of TC due to PTC and a reduction in mortality ( 15 , 16 ).

In 1988, with the promulgation of the current Federal Constitution, access to health, through a universal public health care system became a social right. Law 8,080/1990 ( 17 ), in turn, regulated the Public Health System (SUS), having as main principles and guidelines: universality of access at all levels of health care; equality in assistance, without prejudice and privilege of any kind; integrality of assistance; community participation; and political-administrative decentralization ( 18 ). Although the Constitution inaugurates a SUS in which public health actions and services are expected to be organized in an integrated, regionalized and hierarchical manner, it also considers that health care is free to the private sector. In addition, it was defined that when the availability of own resources was not sufficient to guarantee assistance coverage to the population of a certain area, SUS could resort, through contracts and agreements, to services provided by the private initiative. Thus, beyond SUS, Brazilian health system has other two subsectors: the private health insurance subsector or supplementary health (SH), in which private and public companies offer to their employees health plans and insurance cover, and the private health subsector (PHS), which provides out-of-pocket hospital and ambulatory services ( 19 ).

The objective of the present study was to provide data about the diagnosis and treatment of nodular thyroid disease in the northeastern region of the State of São Paulo (SP) in relation to the types of health care, comparing the SUS, the SH and the PHS, assess the performance of cytologic analysis as a screening test for TC and determine the prevalence of TC in the study sample.

## SUBJECTS AND METHODS

The Regional Health Department of Franca – DRS VIII, located in the northeastern region of the State of São Paulo, covers 22 municipalities with a population of 649,607, according to the 2010 census of the Brazilian Institute of Geography and Statistics (IBGE) ( 20 , 21 ). The data of the present study was obtained in 2014, when 35.7% of the population residing in DRS VIII was covered by the SH while 64.3% was covered by the SUS ( 22 ). Because of limitations in health database systems, data about the percentage of the population attended by PHS was not obtained.

Data of patients with TNs submitted to FNAB from January 1, 2014, through December 31, 2014, were collected at the three pathology services located in the municipality of Franca. These services handle all cytological and histological analyses of DRS VIII, which are performed by the same pathologists.

### Statistical analysis

Descriptive analyses consisted of mean, standard deviation, median and range for numerical variables, and percentage and frequency for categorical variables. Numerical variables were compared using the analysis of variance (ANOVA) with Bonferroni *post hoc* test, Kruskal-Wallis non-parametric test and Dunn *post hoc* test. Categorical variables were compared using the Chi-Square test. Statistical analysis of the data was conducted with the use of SPSS software, version 22.0 (IBM, Chicago, USA), and the level of significance was set at p < 0.05.

The performance of the cytologic analysis was evaluated with the calculation of sensitivity, specificity, accuracy, positive predictive value (PPV), negative predictive value (NPV), positive likelihood ratio (LR+), and negative likelihood ratio (LR-) ( Table 1 ). Bethesda I, III and IV nodules were excluded, and the analysis was made considering Bethesda II as benign and Bethesda V and VI as malignant. PPVs and NPVs were calculated for the prevalence of TC in the sample using Bayes’ theorem ( 23 ), which takes into account the prevalence in question, in addition to the characteristics of the diagnostic test itself, such as sensitivity and specificity values. Confidence intervals were obtained according to methods described in the literature ( 24 – 26 ).

**Table 1:** Measurement of the performance of the cytologic analysis as a diagnostic test, with the exclusion of Bethesda I, III, and IV and inclusion of Bethesda II, V, and VI results

	BENIGN Pathology result	MALIGNANT Pathology result
**NEGATIVE CYTOLOGIC RESULT: Bethesda II**	46	3
**POSITIVE CYTOLOGIC RESULT: Bethesda V and VI**	2	44
**Performance of cytology**	**Estimate**	**95% CI**
S (%)	93.6	81.4-98.3%
Sp (%)	95.8	84.6-99.3%
A (%)	94.7	88.1-98.3%
LR+	22.4	5.71-87.4
LR-	0.06	0.02-0.19
**Prevalence of TC = 9.2%**	**Estimate**	**95% CI**
PPV (%)	69.5	40.7-98.3
NPV (%)	99.3	98.6-100

CI: confidence interval; S: sensitivity; Sp: specificity; PPV: positive predictive value; NPV: negative predictive value; A: accuracy; LR+: positive likelihood ratio; LR-: negative likelihood ratio; TC: thyroid cancer. Benign Pathology result: goiter and colloid cyst, Hashimoto's thyroiditis, adenomatous goiter, follicular Hürthle cell adenoma; Malignant Pathology result: papillary thyroid carcinoma; follicular thyroid carcinoma; medullary thyroid carcinoma.

Overall, 210 nodules were identified in the pathological specimens of the 125 surgical patients. In 162 of these nodules, we were able to verify the cytologic report against the pathology report to estimate the risk of malignancy according to the 2017 Bethesda system ( Table 2 ). We excluded from this analysis 48 nodules with size and location data inconsistent between both reports. It should be pointed out that the collection of the data in the present study occurred before 2016; therefore, encapsulated follicular variant of PTC (EFVPTC) – currently reclassified as NIFTP (noninvasive follicular thyroid neoplasm with papillary-like nuclear features) – was considered to be malignant ( 11 ).

**Table 2:** Risk of malignancy for each cytologic category in the total sample of 162 thyroid nodules compared with the risk of malignancy according to the 2017 Bethesda System, considering NIFPT cases as TCs

	Risk of malignancy in the total sample (n = 162 nodules)	Risk of malignancy according to the 2017 Bethesda System (considering NIFPT = TC)
	%	%
Bethesda I	40.0	5-10
Bethesda II	6.1	0-3
Bethesda III	21.9	10-30
Bethesda IV	25.0	25-40
Bethesda V	88.2	50-75
Bethesda VI	100.0	97-99

NIFPT: Noninvasive follicular thyroid neoplasm with papillary-like nuclear features; TC: thyroid cancer.

The study was approved by the Research Ethics Committee of *Fundação Santa Casa de Misericórdia* of Franca with CAAE ( *Certificado de Apresentação para Apreciação Ética* ) number: 59 358416.2.0000.5438. Waiving of informed consent was requested.

## RESULTS

**Figure 1:**
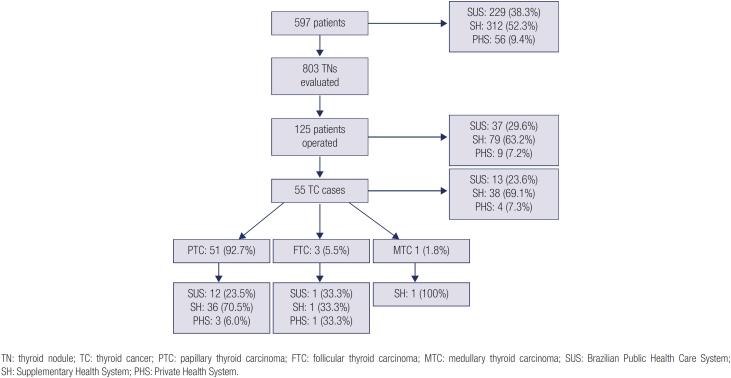
Flowchart of the patient entry into the study.

The study sample consisted of 597 patients, of whom 536 (89.8%) were females and 61 (10.2%) were males. The mean age was 52.5 ± 15.6 years, and the median age was 53 (13–89) years. The mean age was 52.2 ± 15.3 years among females and 55.6 ± 18 years among males. Overall, 229 (38.3%) of the study sample underwent FNAB in the SUS, 312 (52.3%) in the SH, and 56 (9.4%) in the PHS, including 538 (90.1%) who underwent US-guided FNAB and 46 (7.7%) who were submitted to palpation-guided FNAB. In 13 patients (2.2%), the FNAB technique used could not be identified. The percentage of patients submitted to US-guided FNAB was 75.1% in the SUS group, 99.4% in the SH group, and 100% in the PHS group (p < 0.0001). During FNAB, only one TN was aspirated in 442 of the 597 patients (74%), while more than one nodule was aspirated in 155 (26%) patients, yielding a total of 803 cytologic results ( Figure 1 ).

Data on the size of the TNs could not be obtained in 215 cases (26.7%). In the remaining 588 TNs in which the size was known, the mean size was 1.8 ± 1.2 cm, and the median size was 1.5 cm (0.3 – 8 cm). A total of 111 (18.9%) of these nodules were smaller than 1 cm. The percentage of TNs of known size was lower in the SUS (27.3%) compared with the SH (92.6%) and PHS (98.6%; p < 0.01). The mean size of the evaluated TNs was significantly larger in the SUS compared with the SH (2.1 ± 1.2 cm and 1.8 ± 1.1 cm, respectively; p = 0.05). Compared with the SUS and SH, the mean size of the TNs in the PHS (2.0 ± 1.2 cm) was intermediate and not significantly different. The percentage of TNs measuring less than 1 cm was comparable between groups (p = 0.46).

Table 3 shows the distribution of the cytologic results in the SUS (n = 245), SH (n = 486), and PHS (n = 72) groups and for the overall sample of TNs evaluated (n = 803), distributed according to the Bethesda classification system. There was a significant difference (p < 0.001) in the distribution of cytologic results (in percentage) between the types of health care coverage, with a higher percentage of Bethesda I cytology results in the SUS (36.7%) than in the SH (8.4%) and PHS (5.6%), p < 0.05. Bethesda II cytologic results had a lower percentage in the SUS (45.3%) compared with the SH (65.2%) and PHS (65.3%), p < 0.05. For Bethesda IV cytology results, the percentage was lower in the SUS (1.6%) than in the PHS (9.7%; p < 0.05).

**Table 3:** Cytologic results of thyroid nodules in the entire sample and divided according to the type of health care coverage

Cytologic result	SUS n (%)	SH n (%)	PHS n (%)	Total sample n (%)
Bethesda I	90 (36.7) *	41 (8.4)	4 (5.6)	135 (16.8)
Bethesda II	111 (45.3) +	317 (65.2)	47 (65.2)	475 (59.2)
Bethesda III	34 (14.0)	64 (13.2)	9 (12.5)	107 (13.3)
Bethesda IV	4 (1.6) #	21 (4.3)	7 (9.7)	32 (4.0)
Bethesda V	2 (0.8)	17 (3.5)	1 (1.4)	20 (2.5)
Bethesda VI	4 (1.6)	26 (5.4)	4 (5.6)	34 (4.2)
Total	245 (100)	486 (100)	72 (100)	803 (100)

SUS: Brazilian Public Health Care System; SH: Supplementary Health; PHS: Private Health System.

* SUS > SH (p<0.05); SUS > PHS (p<0.05).

+ SUS < SH (p<0.05); SUS < PHS (p<0.05).

# SUS < PHS (p<0.05).

The percentage of Bethesda I cytologic results among patients submitted to US-guided FNAB was greater is SUS (38.3%) compared with SH (12.9%) and PHS (7.1%), p < 0.001. There was no difference between SH and PHS, p = 0.66.

A total of 125 patients (20.9%) underwent thyroidectomy, and TC was diagnosed in 55 of them. A comparison between the 125 patients who underwent surgery versus the 472 who did not undergo this procedure revealed no difference in the proportion of men and women (88.8% women *versus* 11.2% men among the surgical patients and 90% women and 10% men among the nonsurgical patients; p = 0.9) or TN size (1.9 ± 1.4 cm *versus* 1.8 ± 1.1 cm, respectively; p = 0.61). However, the mean age of the surgical patients was significantly lower (48.6 ± 14.5 years) than that of the nonsurgical ones (53.6 ± 15.8 years; p = 0.001). The prevalence of TC in the overall cohort was 9.2%.

The mean time elapsed between the cytologic analysis and surgery was longer in patients evaluated in the SUS (246 ± 187 days, n = 41) compared with those evaluated in the SH (143 ± 128 days, n = 78; p < 0.001). The mean time elapsed between both procedures in the PHS group (n = 6) was 45 ± 22 days. Among patients with Bethesda V and VI cytologic results, the mean time elapsed between evaluation and surgery was also longer in the SUS group (231 ± 241 days, n = 9) than in the SH group (66 ± 47 days, n = 33; p = 0.01). The corresponding mean time in the PHS group (n = 3) was 42 ± 24 days. The mean size of the TNs described in the pathology report was larger in the SUS group (3.1 ± 1.7 cm, n = 35) than in the SH group (2.2 ± 1.8 cm, n = 73; p = 0.001). The corresponding mean size in the PHS group (n = 6) was 1.1 ± 0.7 cm. Eleven patients with benign pathological results for whom the size of the nodule was not available were excluded from this analysis, including 7 in the SUS group and 4 in the SH group. The size of the largest resected malignant TN was greater in the SUS group (2.6 ± 1.7 cm, n = 13) than in the SH group (1.7 ± 1.5 cm, n = 38; p = 0.02). The corresponding mean size in the PHS group (n = 4) was 1.3 ± 0.8 cm.

Of the 55 patients with TC, 46 (83.6%) were females and 9 (16.4%) were males. Their mean age was 46.9 ± 16.1 years and median age was 45 years (20–82 years). Histologically, the resected TCs comprised 51 (92.7%) PTCs, 3 (5.5%) minimally invasive FTCs, and 1 (1.8%) MTC. In 17 (31%) patients with PTC, the largest tumor measured ≤ 1 cm (microPTC).

Among the 51 patients with PTC, the tumor was unifocal in 30 (58.8%) and multifocal in 21 (41.2%) cases. From the total of 79 PTCs resected, 48 (60.7%) were classical variants, 23 (29.1%) were follicular variants (FVPTC), 7 (8.9%) were oxyphilic variants, and 1 (1.3%) was a tall cell variant. The mean and median sizes of the PTCs, which could be analyzed in 77 tumors, were 1.3 ± 1.1 cm and 1 cm (0.15–5.5 cm), respectively. Among the 23 FVPTC, 7 (30.5%) were EFVPTC, 10 (43.5%) were nonencapsulated, and 6 (26%) had no description about encapsulation. The 7 EFVPTCs occurred in 4 patients and represented 7.3% of the TC cases in the study sample.

The calculated performance of cytology was: sensitivity 93,6%, specificity 95,8%, accuracy 94,7%, PPV 69,5%, NPV 99,3%, LR+ 22,4 and LR- 0,06 ( Table 1 ).

## DISCUSSION

The objective of evaluating a TN is to identify and correctly treat those nodules that impose a health risk to the patient. Guidelines by the American Thyroid Association (ATA) published in 2015 ( 1 ) and by the American Association of Clinical Endocrinologists/American College of Endocrinology/Associazione Medici Endocrinologi (AACE/ACE/AME) published in 2016 ( 3 ), along with updates like the Bethesda System 2017 ( 11 ) and the 8^th^ edition of the American Joint Committee on Cancer/Tumor-Node-Metastasis Staging System (AJCC/TNM) published in 2018 ( 27 ), synthesize recent conceptual and management changes related to TNs.

In the present study including 597 patients, the proportion of patients submitted to FNAB in the SUS and the SH (38.3% versus 52.3%, respectively) differed from the proportion of patients with SUS and SH coverage in the DRS VIII region (64.3% and 35.7%, respectively). This inconsistency was possibly due to unequal access to consultations and specialized tests between the public and private systems and to a greater concentration of specialized physicians in the private health sector ( 28 ), which favors timely diagnosis, evaluation, and treatment of patients with TNs with SH coverage.

Of the TNs of known size evaluated in this study (n = 588), 18.9% were smaller than 1 cm. According to recent guidelines ( 1 , 3 ), TNs smaller than 1 cm do not require evaluation with FNAB, except in specific situations like those of lesions with a subcapsular or paratracheal location, presence of suspected lymph nodes or extrathyroid extension, personal or family history of TC, or suspicious clinical findings like dysphonia ( 3 ). By applying these recommendations to the sample, about 111 TNs would not be evaluated with FNAB, thus reducing costs, demand for consultations and tests, and decreasing the patients’ anxiety level.

The distribution of the FNAB results according to Bethesda categories ( Table 3 ) indicates that 65.9% of the patients had Bethesda II, V, and VI cytologic results, *i.e.* , categories in which the cytologic analysis typically has a better performance in distinguishing between benign and malignant lesions. On the other hand, 34.1% of the results were nondiagnostic (Bethesda I) or indeterminate (Bethesda III and IV). In other published series, the corresponding percentages were 32.4% ( 29 ) and 20.1% ( 9 ).

Bethesda I nodules represented 16.8% of all cytologic results in our study (n = 135), with a higher percentage of this diagnosis established in the SUS (36.7%) compared with the SH (8.4%) and PHS (5.6%; p < 0.05) groups ( Table 3 ). In large series of cytological studies, the Bethesda I category represented 2–16% of the cases ( 30 ), although the literature reports rates below 10% when cystic lesions are excluded ( 3 , 11 ). These rates are influenced by the structural aspects of the aspirated TN (cystic or partially cystic), FNAB technique (needle diameter, collection of the sample by capillarity or by aspiration) ( 31 , 32 ), preparation of the smear on the slide, use or not of cell blocks ( 29 ), and experience of the professional collecting the sample ( 33 ). The use of US-guided FNAB decreases the rates of Bethesda I results compared with palpation-guided FNAB ( 1 , 34 ). In the present study, 90.1% of the FNABs were guided by US, and important differences were observed depending on the type of health care coverage (SUS 75.1%, SH 99.4%, PHS 100%; p < 0.0001). The differences found in the percentage of Bethesda I results in US-guided FNAB suggests the occurrence of technical problems with the collection and/or fixation of the material in the SUS and underscores the need for revision of the employed technique.

Regarding the group of 125 patients who underwent surgery, some important differences were detected between types of health care coverage, such as time elapsed between the cytologic diagnosis and surgery, the size of the nodule that based the surgical recommendation, and the size of the malignant nodule; all these parameters were greater in the SUS compared with the SH group. This may be explained by unequal access to consultations and specialized tests in the public and private systems in Brazil and by issues involving continued medical education.

The prevalence of TC in the present sample was 9.2%, which is comparable to the prevalence rates of TC reported in the literature (5–15%) ( 1 , 2 ), with PTCs comprising most of all TCs (92.7%). Most TCs (76.4%) were treated in the SH and PHS and 23.6% of them were treated in the SUS. A similar trend has been reported by Cordioli and cols. ( 15 ) in Florianópolis, where only 11.7% of the cases of TC diagnosed in 2005 were from the public system, and by Veiga and cols. ( 16 ) in São Paulo, where 75% of such cases were diagnosed in the private sector. Seventeen patients with microPTC ( 31 % of the TC cases) were treated surgically. Recent data suggest that clinical monitoring can be used as the initial management in low-risk microPTCs ( 1 ). In a clinical trial by Miyauchi and cols., 8% of unresected microPTCs grew in size and only 3.8% developed lymph node metastasis over a 10-year follow-up period. The cost of surgery as the initial management was 4.1 times higher than the cost of clinical monitoring in the Japanese health care system ( 35 ).

In the present study, 4 patients had a diagnosis of EFVPTC. It is important to point out that the reports of the patients included in the present study were issued during a period that preceded the reclassification of EFVPTC to NIFTP and, therefore, did not follow the diagnostic criteria defined in 2016 ( 13 ). If we assume that these patients had NIFPT, 7.3% of the TC cases in our series would have received a different classification and would no longer be diagnosed as cancer.

Regarding the risk of malignancy, 4 of the 10 TNs repeatedly classified as Bethesda I and submitted to surgery were malignant ( Table 2 ). This malignancy rate is higher than the one estimated by the Bethesda classification system (5–10%) but close to that observed in surgically resected Bethesda I nodules (9–32%) ( 11 ). The calculation of the risk of malignancy in Bethesda I nodules is known to be complex and to overestimate the risk among resected TNs.

The risk of malignancy in Bethesda II nodules (6.1%) was higher than that estimated by the Bethesda classification system (0–3%), with 3 patients with PTC detected among 49 resected TNs with a Bethesda II cytology.

Indeterminate cytology results showed risks of malignancy of 21.9% (Bethesda III) and 25% (Bethesda IV), which are within the values estimated by the Bethesda classification system, *i.e.* , 10–30% and 25–40%, respectively. This is an important result of the present study. Professionals involved in the care of patients with TNs should be aware of these statistics and inform them to the patients, since they may help managing decisions in terms of choosing between conservative and surgical treatment and defining the extent of the thyroidectomy.

Bethesda V and VI cytologic results had a high risk of malignancy (88.2% and 100%, respectively), confirming that the surgery is indeed the most appropriate management for this TN category.

In this study, cytologic analysis showed a sensitivity of 93.6%, specificity of 95.8%, and accuracy of 94.7% in the detection of TCs when Bethesda III and IV categories were excluded, and was considered a good screening test for TC. In published series, the sensitivity and specificity of the cytologic analysis range from 65–98% and 73–100%, respectively ( 9 ). This wide variation is due to differences among studies in the categorization of Bethesda III and IV cytologic results and inclusion of adenomas and NIFTP nodules, a fact that hinders comparison with the present results.

An important limitation of the present study was the unavailability of the patients’ clinical information, US data of the evaluated TNs, and tests for postoperative staging of the TC cases. This occurred due to lack of interoperability between health database systems, so the only converging point of data on TNs and TCs occurred at the pathology services. An easy fill evaluation form about TN and TC could be inserted in a more integrated health database.

In conclusion, the present study detected important differences in the treatment of TNs between the public and private health care sectors in the northeastern region of the State of São Paulo (DRS VIII), indicating a need for management of issues such as access to consultations and specialized tests, and effective application of recent guidelines. The prevalence of TCs in the study sample was 9.2% (31% were ≤ 1 cm), and 23.6% of the TC cases were treated surgically in the SUS. The cytologic analysis showed a sensitivity of 93.6%, specificity of 95.8%, and accuracy of 94.7% in the detection of TCs when Bethesda III and IV categories were excluded, reinforcing its role as a good screening method for Bethesda II, V, and VI nodules.
